# Raptor couples mTORC1 and ERK1/2 inhibition by cardamonin with oxidative stress induction in ovarian cancer cells

**DOI:** 10.7717/peerj.15498

**Published:** 2023-06-07

**Authors:** Yanting Zhu, Shifeng Wang, Peiguang Niu, Huajiao Chen, Jintuo Zhou, Li Jiang, Danyun Li, Daohua Shi

**Affiliations:** Department of Pharmacy, Fujian Maternity and Child Health Hospital, College of Clinical Medicine for Obstetrics & Gynecology and Pediatrics, Fujian Medical University, Fuzhou, China

**Keywords:** Cardamonin, Ovarian cancer, Oxidative stress, Raptor, ERK1/2

## Abstract

**Background:**

A balance on nutrient supply and redox homeostasis is required for cell survival, and increased antioxidant capacity of cancer cells may lead to chemotherapy failure.

**Objective:**

To investigate the mechanism of anti-proliferation of cardamonin by inducing oxidative stress in ovarian cancer cells.

**Methods:**

After 24 h of drug treatment, CCK8 kit and wound healing test were used to detect cell viability and migration ability, respectively, and the ROS levels were detected by flow cytometry. The differential protein expression after cardamonin administration was analyzed by proteomics, and the protein level was detected by Western blotting.

**Results:**

Cardamonin inhibited the cell growth, which was related to ROS accumulation. Proteomic analysis suggested that MAPK pathway might be involved in cardamonin-induced oxidative stress. Western blotting showed that cardamonin decreased Raptor expression and the activity of mTORC1 and ERK1/2. Same results were observed in Raptor KO cells. Notably, in Raptor KO cells, the effect of cardamonin was weakened.

**Conclusion:**

Raptor mediated the function of cardamonin on cellular redox homeostasis and cell proliferation through mTORC1 and ERK1/2 pathways.

## Introduction

Epithelial ovarian cancer (EOC) is one of the leading causes of cancer-related mortality among females due to the high risk of metastasis and recurrence (Torre et al., 2018). The standard treatment for ovarian cancer is optimal debulking combined with platinum-based chemotherapy, while the majority of patients eventually develop chemotherapy resistance (Feng et al., 2021). Therefore, there is an urgent need to find high-efficiency and low-toxicity therapeutic drugs to improve chemotherapy sensitivity for ovarian cancer.

A balance on nutrient supply and redox homeostasis is required for cell survival. Under the condition of ischemia and hypoxia, malignant cells acquire metabolic adaptation, which leads to overproduction of reactive oxygen species (ROS) (Panieri & Santoro, 2016). Changes in intracellular ROS levels are inseparable from the tumor cell growth and exert discrepant impacts on cancer cell survival (Hayes, Dinkova-Kostova & Tew, 2020). Appropriate increase of ROS promotes the proliferation and migration of tumor cells, whereas excessive ROS induces oxidative stress and cell death by damaging to DNA, protein, mitochondria and endoplasmic reticulum. As a result, antioxidants in cancer cells are largely synthesized. It has been reported that the first-line chemotherapeutics such as cisplatin and paclitaxel commonly generated excessive of ROS in ovarian cancer cells, thereby promoting cell death (Kleih et al., 2019). However, the elevated capacity of anti-oxidative in tumor cells can neutralize the oxidative stress and render cells to chemotherapy resistance (Yang et al., 2018). Multiple pathways have been reported to be involved in the regulation of ROS production and elimination. For example, the cross-talk between AMPK and AKT was proved to be related with ROS regulation and cancer progression (Zhao et al., 2017). Although numerous of studies have devoted to uncovering the potential mechanisms of ROS regulation, the optimal targets of ROS remain unknown.

The MAPK signaling pathway participates in several cellular processes and can be activated by ROS (Chang et al., 2021). There are four members in MAPK family, including extracellular signal-regulated kinases (ERKs), c-Jun N-terminal kinases (JNKs), extracellular regulated protein kinases 5 (ERK5) and p38. Among them, ERK1/2 was well studied in cell signal transduction regulating and high activation of ERK1/2 was related to the occurrence and development of cancer (Guo et al., 2020). On the contrary, inhibition of ERK1/2 was proved to suppress tumor cell epithelial-mesenchymal transition (EMT) and induce apoptosis in ovarian cancer cells (Shi et al., 2020). ERK1/2 inhibition could also aggravate intracellular oxidative stress and inflammation by promoting intracellular ROS accumulation (Wang et al., 2021a). In addition, ERK/p38 MAPK combined with the NF-κB pathway was involved in cell proliferation of hepatocellular carcinoma in a ROS-dependent manner (Yuan et al., 2019). Notably, it was reported that ROS generation induced by ERK might partially contribute to cell apoptosis (Park et al., 2017).

The atypical serine/threonine kinase mTOR is a key regulator of cell growth and metabolism, who contains two structurally and functionally distinct complexes, mTOR complex 1 (mTORC1) and mTORC2. Regulatory-associated protein of TOR (Raptor) is an mTOR-binding partner that also acts as a scaffold protein to p70 S6 kinase 1 (p70S6k1) and eukaryotic initiation factor 4E-binding protein 1 (4EBP1). Raptor is indispensable for mTOR-catalyzed phosphorylation of downstream translation regulators. Upon stimulation, mTOR is activated and regulates several intracellular physiological processes through a variety of pathways (Kim & Guan, 2019). Importantly, high activation of mTOR in tumor cells will reprogram the cell metabolism by altering the availability of metabolic enzymes (Liu & Sabatini, 2020). In mTOR hyper-activated cells, decreased level of ROS was observed, which subsequently led to uncontrolled cell growth and drug resistance (Wang et al., 2021c). Inhibition of mTOR could inhibit the cell proliferation and migration in ovarian cancer (Fan et al., 2021). Targeting the PI3K/AKT/mTOR signaling pathway could regulate cell fate by inducing oxidative stress damage in glioma cells (Li et al., 2020). Rapamycin, a classical inhibitor of mTORC1, inhibited the tumor cells growth through inducing cell oxidative stress (Zimmerman et al., 2020). Deregulated of PI3K/AKT/mTOR and Raf/MEK/Erk signaling pathways were observed simultaneously in human cancer cells (Wilhelm et al., 2004; Will et al., 2014), and the subsequent excessive production of ROS has been reported to be associated with apoptosis and autophagy (Niu et al., 2015a; Pan et al., 2015). In addition, simultaneous inhibition of mTOR and ERK1/2 could further increase the accumulation of ROS in tumor cells (Jasek-Gajda et al., 2020), suggesting a synergistic effect of combined mTOR and ERK1/2 inhibitors for cancer therapy. Recently, drugs that targeting nodes of these two pathways are being investigated, providing a promising therapeutic strategy for chemotherapy-resistant cancer (Chen et al., 2018). However, the crosstalk between the two pathways remains unclear.

Cardamonin is a chalcone and its anti-cancer role has been extensively studied. Our previously results showed that cardamonin have a chemo-preventive effect on non-small-cell lung cancer, Lewis lung cancer, breast cancer and ovarian cancer (Niu et al., 2015b; Shi et al., 2018b; Tang et al., 2014). We also demonstrated that the pharmacological activities of cardamonin are related to mTORC1 inhibition partly through down-regulating the expression of Raptor (Liao et al., 2010; Niu et al., 2013; Shi et al., 2018b; Zheng et al., 2010; Zhu et al., 2021). Notably, cardamonin exerts bidirectional regulation of redox homeostasis under different circumstance. Cardamonin protected the heart from oxidative damage and inflammatory injury by activating Nrf2-related cytoprotective system in cardiac muscle cells (Qi et al., 2020), whereas cardamonin suppressed tumor cell growth by inducing oxidative stress through NF-κB/mTOR and HIF-1α pathways (Jin et al., 2019; Ruibin et al., 2020). Therefore, further studies are needed to investigate the mechanism by which cardamonin regulates oxidative homeostasis. In the present study, we attempted to explore the correlation between the mechanism of cardamonin-induced oxidative stress and ERK1/2 and mTOR pathways in ovarian cancer cells.

## Materials and Methods

### Reagents and chemicals

Cardamonin (no 110763, purity >99%; National Institutes for Food and Drug Control, Beijing, China), rapamycin (Sigma-Aldrich Co., St Louis, MO, USA), MHY1485 (HY-B0795; MedChemExpress, Monmouth Junction, NJ, USA), AZD0364 (S8708; Seleck Chemicals, Houston, TX, USA), N-Acetyl-L-cysteine (S26121; Yuanye Biological Co., Shanghai, China), and glutamic acid (SG8540-200; Solarbio Co., Beijing, China) were dissolved in dimethyl sulfoxide (DMSO; Sigma-Aldrich Co., St Louis, MO, USA) and stored at 4 °C. CCK8 kit was purchased from Wabcan Co. and Reactive Oxygen Species Assay Kit was purchased from Beyotime Institute of Biotechnology (Jiangsu, China). Antibodies against mTOR, p-mTOR (Ser2481), Raptor, p-p44/42 MAPK (ERK1/2), p44/42 MAPK (ERK1/2), S6K1, p-S6K1 (Thr389), β-actin and the secondary antibodies were purchased from Cell Signaling Technology (Danvers, MA, USA).

### Cell culture

Human ovarian cancer SKOV3 and A2780 cells were obtained from the Boster Biological Technology Co., Ltd (Wuhan, Hubei, China). SKOV3 cell line, Raptor knockout SKOV3 cell line (previously constructed by CRISPR/Cas 9) and A2780 cell line were prepared with appropriate medium containing 10% fetal bovine serum (FBS) and 1% penicillin-streptomycin. Cells were incubated in 37 °C constant temperature incubator with 5% CO_2_.

### Cell viability analysis

Cell viability was determined by CCK8 assay. Cells were seeded into a 96-well plate with about 1 × 10^3^ cells per well, and then treated with indicated drugs for 24 h. The supernatant was discarded carefully, and then 10% CCK8 reagent was added to each well. Cells were continuing incubated for 1 h. The absorbance was determined at 450 nm by a microplate reader (Model 1680; Bio-Rad Laboratories Inc., Hercules, CA, USA).

### Wound healing assay

Cells were seeded in 6-well plates and a “wound” was inflicted by a sterile pipette tip when cells grown into a confluent monolayer. Then cells were washed with PBS twice and incubated with drugs for 24 h. Photos were taken at ×100 magnification at 0 and 24 h after drugs treatment, and the wound closure was measured by ImageJ program.

### Flow cytometry

Cells were seeded in 6-well plates, and treated with drugs for 24 h. Then cells were incubated with 10 μM dichlorodihydro-fluorescein diacetate (DCFH-dA) in a constant temperature shaker at 37 °C for 30 min in the dark. A density of 1 × 10^6^ cells were collected and re-suspended in PBS, and the Dichlorofluorescein diacetate (DCF-dA) fluorescence intensity was determined by flow cytometry within 2 h. The mean fluorescence intensity (MFI) was used to quantify the levels of ROS.

### Tandem mass tag (TMT)-labeling proteomic analysis

Samples were ground into cell powder and sonicated by a high-intensity ultrasonic processor with lysis buffer (8 M urea, 1% Protease Inhibitor Cocktail). Removed the remaining debris by centrifugation and collected the supernatant, following by determining the protein concentration with a BCA kit. Digested the protein solution with dithiothreitol and alkylated the protein with iodoacetamide at room temperature in darkness. Diluted the protein sample and digested proteins by trypsin. TMT labelling was conducted according to the manufacturer’s protocol from the TMT kit and as described previously (Zhang et al., 2019), then LC-MS/MS analysis was performed by an EASY-nLC 1000 UPLC system, and the Maxquant search engine (v.1.5.2.8) was used to process the MS/MS data.

### Western blotting analysis

Cells were washed twice with ice-cold PBS after treated with indicated drugs for 24 h and re-suspended in lysis buffer for 30 min. Lysates were centrifuged and the supernatant was collected. Protein concentration was measured by a BCA kit. Proteins were then separated on sodium dodecyl sulfate-polyacrylamide gel electrophoresis, followed by transferring to polyvinylidene fluoride membranes. The membranes were blocked in 5% bovine serum albumin for 1 h and then incubated with indicated antibodies at 4 °C overnight. Washed the membranes with Tris-buffered saline containing 0.1% Tween 20 for three times and then incubated the membranes with the appropriate secondary antibodies for 1 h. Washed the membranes for another three times, and added the HRP-enhanced chemiluminescence reagents to react with the secondary antibodies, and then developed the bands on the membranes by autoradiography (KODAK Film, Shanghai, China). Finally, protein bands were quantified by BioImaging Systems.

### Statistical analysis

Statistical analysis was performed by using the SPSS 21.0 software (SPSS, Inc., Chicago, IL, USA), and all experimental data were expressed as mean ± SD. Differences between two groups were evaluated by the Student’s t-test and multi-component comparison were performed by one-way analysis of variance (one-way ANOVA). *P* < 0.05 was considered to be statistically significant.

## Results

### Cardamonin inhibited the cell viability and migration through induction of oxidative stress in ovarian cancer cells

**Figure 1:**
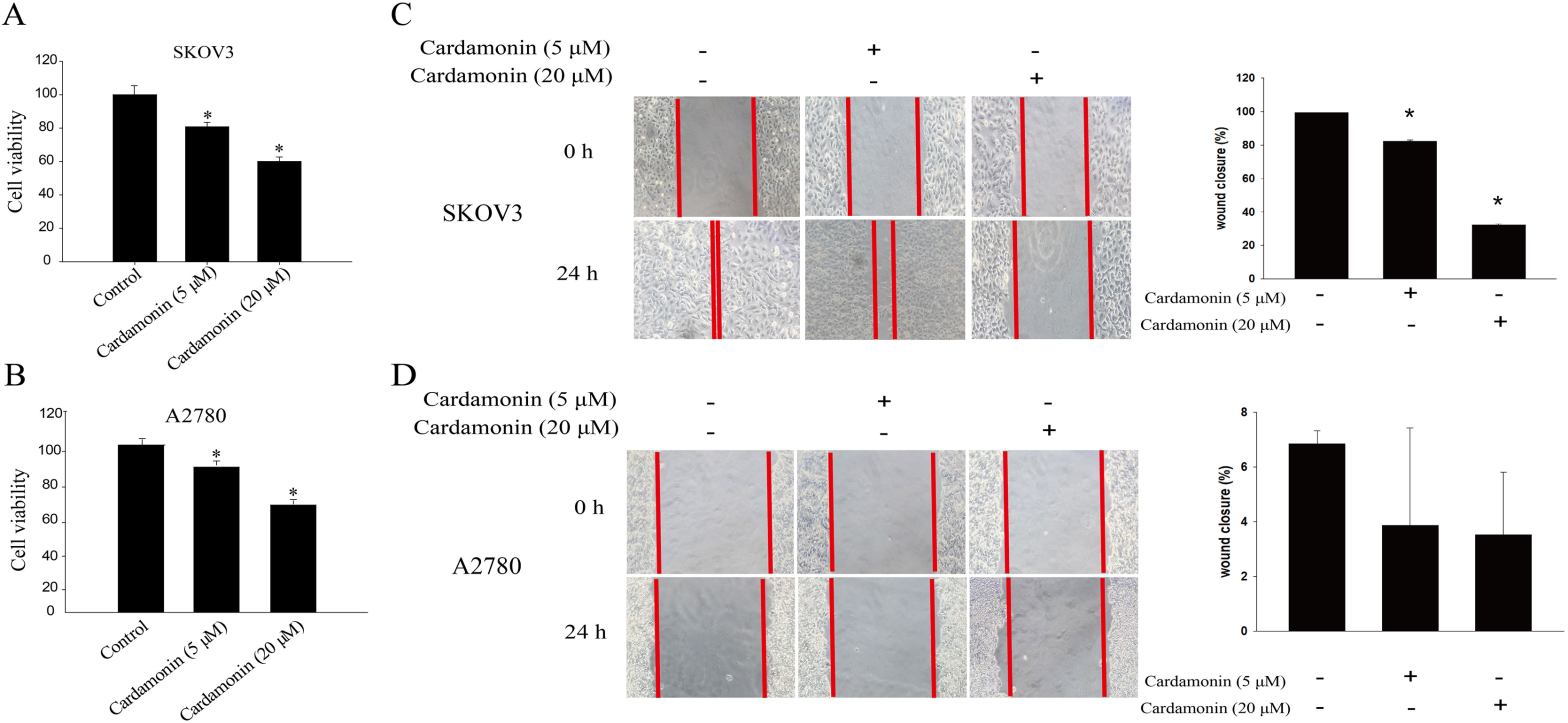
Cardamonin inhibited the cell viability and migration of SKOV3 cells and A2780 cells. Cells were treated with cardamonin (5, 20 μM) for 24 h, respectively. (A) The cell viability of SKOV3 cells. (B) The cell viability of A2780 cells. (C) The migration of SKOV3 cells. (D) The migration of A2780 cells. Mean ± SD, *n* = 3, *P < 0.05 *vs* control group.

**Figure 2:**
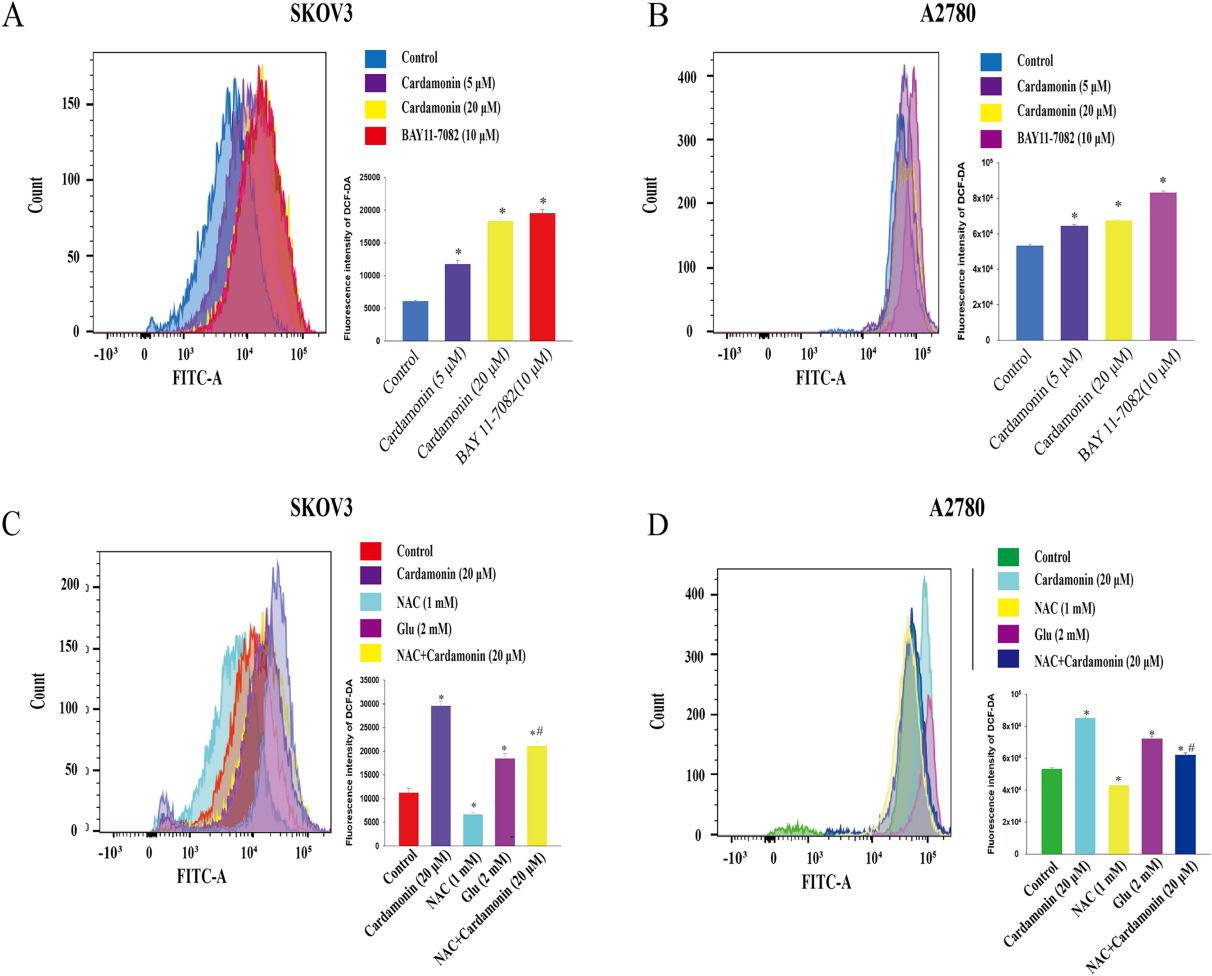
Cardamonin induced ROS accumulation in ovarian cancer cells. Cells were treated with cardamonin (5, 20 μM) or BAY11-7082 (10 μM) for 24 h, respectively. (A) The ROS level in SKOV3 cells. (B) The ROS level in A2780 cells. Cells were treated with cardamonin (20 μM), NAC (1 mM) or Glu (2 mM) for 24 h, respectively. For further study, cells were pretreated with NAC (1 mM) in SKOV3 and A2780 cells for 1 h, and followed by cardamonin (20 μM) treatment for 24 h, respectively. (C) The ROS level in SKOV3 cells. (D) The ROS level in A2780 cells. Mean ± SD, *n* = 3, *P < 0.05 *vs* control group, *^#^P* < 0.05 *vs* Cardamonin (20 μM) group.

**Figure 3:**
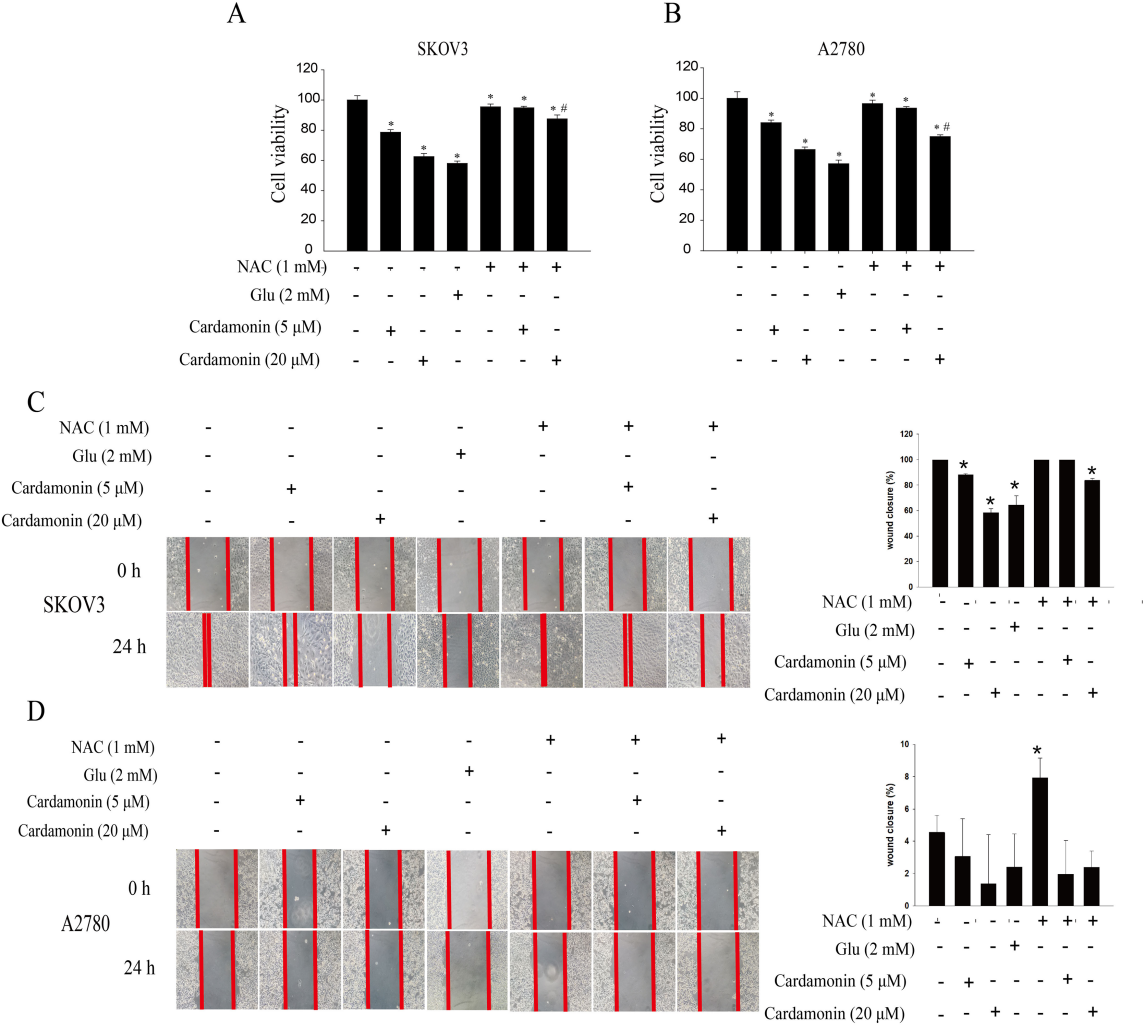
Cardamonin inhibited the cell viability and migration of ovarian cancer cells by induction oxidative stress. Cells were treated with cardamonin (5, 20 μM), NAC (1 mM) or Glu (2 mM) for 24 h, respectively. For further study, cells were pretreated with NAC (1 mM) in SKOV3 and A2780 cells for 1 h, and followed by cardamonin (5, 20 μM) treatment for 24 h, respectively. (A) The cell viability of SKOV3 cells. (B) The cell viability of A2780 cells. (C) The migration of SKOV3 cells. (D) The migration of A2780 cells. Mean ± SD, *n* = 3, *P < 0.05 *vs* control group, *^#^P* < 0.05 *vs* Cardamonin (20 μM) group.

To verify whether the inhibitory of cardamonin on cell viability and migration was related to induction of oxidative stress, A2780 and SKOV3 cells were treated with different doses of cardamonin. Cell viability and migration were measured by CCK8 kit assay and wound healing assay, respectively. Results showed that cardamonin inhibited the cell viability of both A2780 and SKOV3 cells in a dose-dependent manner (Figs. 1A and 1B). In addition, cardamonin significantly inhibited the migration of SKOV3 cells. However, A2780 cells appeared to grow in superposition, with only a slight change in cell migration rate after 24 h of culture, and cardamonin had little inhibitory effect on its migration (Figs. 1C and 1D). Next, we further studied the effect of cardamonin on intracellular ROS level and cell death. Results showed that intracellular ROS was markedly increased by cardamonin (5, 20 μM) and BAY11-7082 (10 μM, a positive agent). We observed a little stronger effect of cardamonin in ROS induction on SKOV3 cells than that on A2780 cells (Figs. 2A and 2B). Then the reactive oxygen scavenger N-acetyl-L-cysteine (NAC) and the oxidative stress inducer glutamate (Glu) were used to further confirm whether the inhibitory of cardamonin on ovarian cancer was related to ROS production. Cells were pretreated with NAC or Glu 1 h before cardamonin treatment. NAC reduced but Glu promoted the accumulation of intracellular ROS content. In addition, the effect of cardamonin on generation of ROS was weakened by NAC pretreatment (Figs. 2C and 2D). Accordingly, NAC treated alone showed little effect (a moderately decreased) on cell viability, while Glu markedly inhibited the cell viability. Furthermore, cell viability was partly restored by NAC pretreatment in cardamonin groups (Figs. 3A and 3B). The inhibitory of cardamonin on the migration of cancer cells was also proved to be related with ROS accumulation. The results of the wound healing assay showed that Glu significantly inhibited while NAC promoted the migration, respectively; in addition, NAC partially abolished the inhibitory of cardamonin on cell migration in SKOV3 cells. Nevertheless, the migration was not affected by any drugs except for NAC in A2780 cells (Figs. 3C and 3D).

### Cardamonin inhibited the ERK1/2 pathway

**Figure 4:**
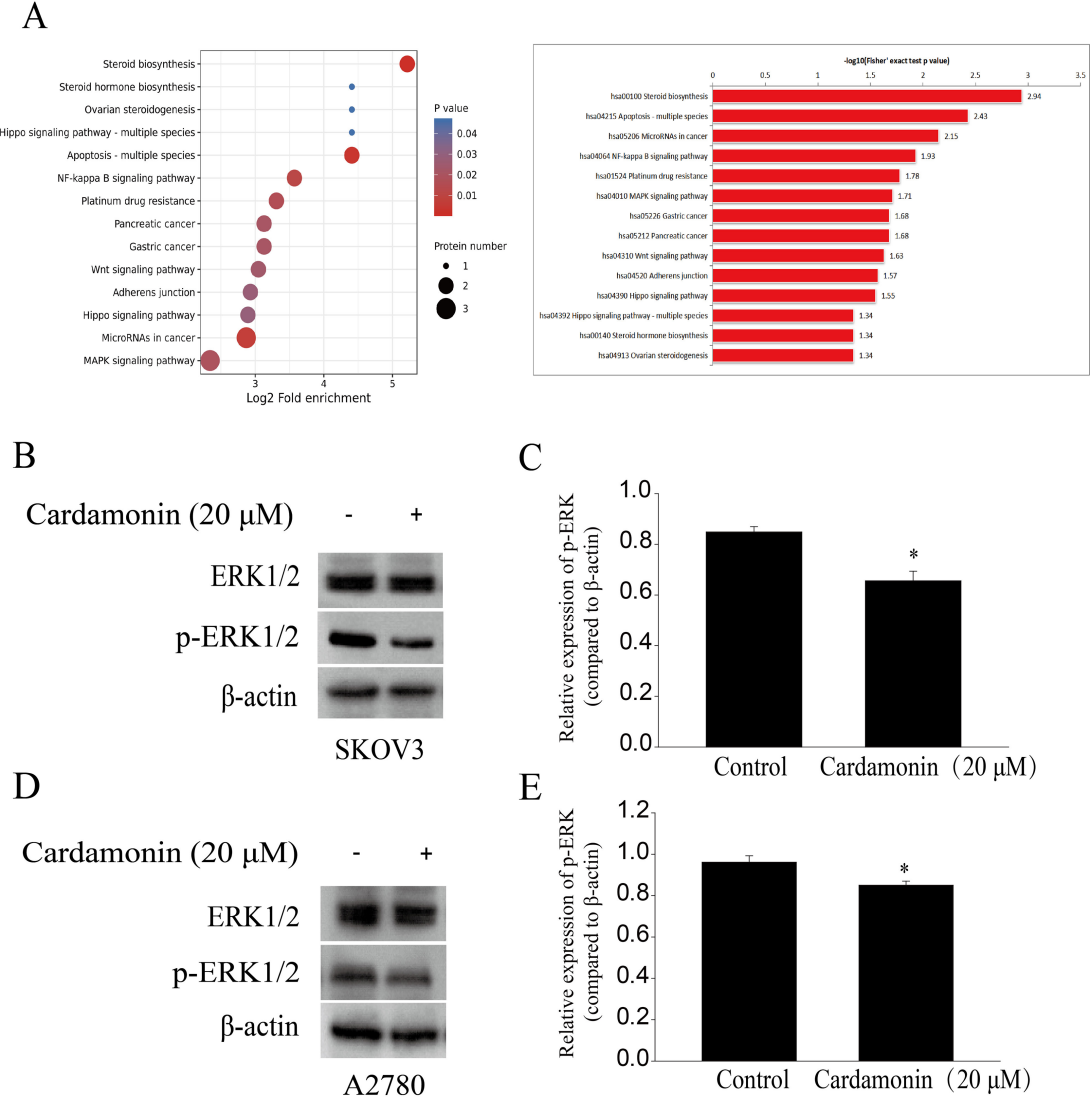
Cardamonin inhibited ERK1/2 pathway activity. Cells were treated with cardamonin (20 μM) for 24 h, and then total protein was extracted for proteomic analysis and Western blotting assay. (A) KEGG pathway enrichment analysis of the DAPs under cardamonin treatment. (B) The protein bands of p-ERK1/2 and actin in SKOV3 cells. (C) The relative density ratios of p-ERK1/2 protein were normalized to actin in SKOV3 cells. (D) The protein bands of p-ERK1/2 and actin in A27803 cells. (E) The relative density ratios of p-ERK1/2 protein were normalized to actin in A2780 cells. For all panels, error bars are presented as the mean ± SD, *n* = 3, *P < 0.05 *vs* control group.

To characterize proteomic alterations associated with cardamonin (20 μM), we conducted comparative proteomic analyses in SKOV3 cells. The InterProScansoft was used to annotate the GO functions of the proteins and the protein sequence alignment method and Kyoto Encyclopedia of Genes and Genomes (KEGG) database was used to annotate the protein pathways. The results showed that the MAPK signaling pathway, which was reported previously in relation with redox homeostasis regulation, was significantly down-regulated by cardamonin (Fig. 4A). We also confirmed that cardamonin markedly decreased the phosphorylation ERK1/2 without affecting the total protein levels by Western blotting analysis both in SKOV3 and A2780 cells (Figs. 4B–4E).

### Cardamonin inhibited the mTORC1 pathway

**Figure 5:**
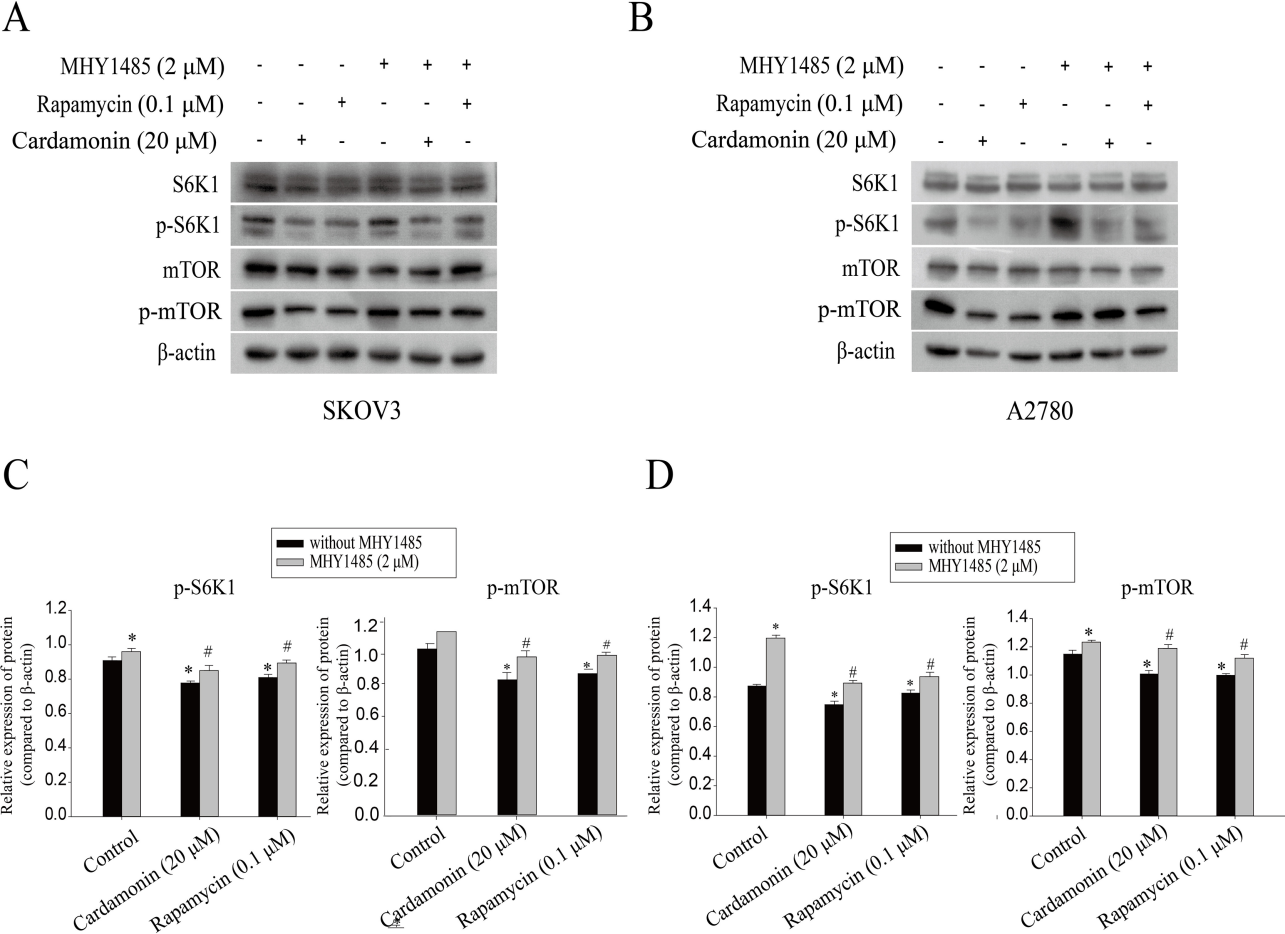
Cardamonin inhibited mTORC1 pathway activity. Cells were treated with cardamonin (20 μM), rapamycin (0.1 μM) or MHY1485 (2 μM) for 24 h, respectively. For further study, cells were pretreated with MHY1485 (2 μM) in SKOV3 and A2780 cells for 1 h, and followed by cardamonin (20 μM) or rapamycin (0.1 μM) treatment for 24 h, respectively. Then, total protein was extracted for Western blotting analysis. (A) The protein bands of p-mTOR, mTOR, p-S6K1, S6K1 and actin in SKOV3 cells. (B) The protein bands of p-mTOR, mTOR, p-S6K1, S6K1 and actin in A27803 cells. (C) The relative density ratios of p-S6K1 and p-mTOR protein were normalized to actin in SKOV3 cells. (D) The relative density ratios of p-S6K1 and p-mTOR protein were normalized to actin in A2780 cells. For all panels, error bars are presented as the mean ± SD, *n* = 3, *P < 0.05 *vs* non-specific control (NC) group, *^#^P* < 0.05 *vs* non-MHY1485 pretreatment groups.

Our previous study showed that cardamonin inhibited the expression of hypoxia-inducible factor-α (HIF-α) and vascular endothelial growth factor (VEGF), under CoCl_2_-mimicked hypoxia conditions, which was partially correlated with mTOR inhibition (Xue et al., 2016). The role of mTOR in oxidative stress regulated by cardamonin was then further investigated. In line with previous studies, cardamonin inhibited the phosphorylation of S6K1 at Thr389 and mTOR at Ser2448, without affecting the total protein expression. In addition, the inhibitory of cardamonin on the phosphorylation of mTOR and S6K1 was weakened by MHY1485 (a classic mTOR agonist) (Figs. 5).

### Raptor coupled mTORC1 and ERK1/2 inhibition by cardamonin with oxidative stress induction in ovarian cancer cells

**Figure 6:**
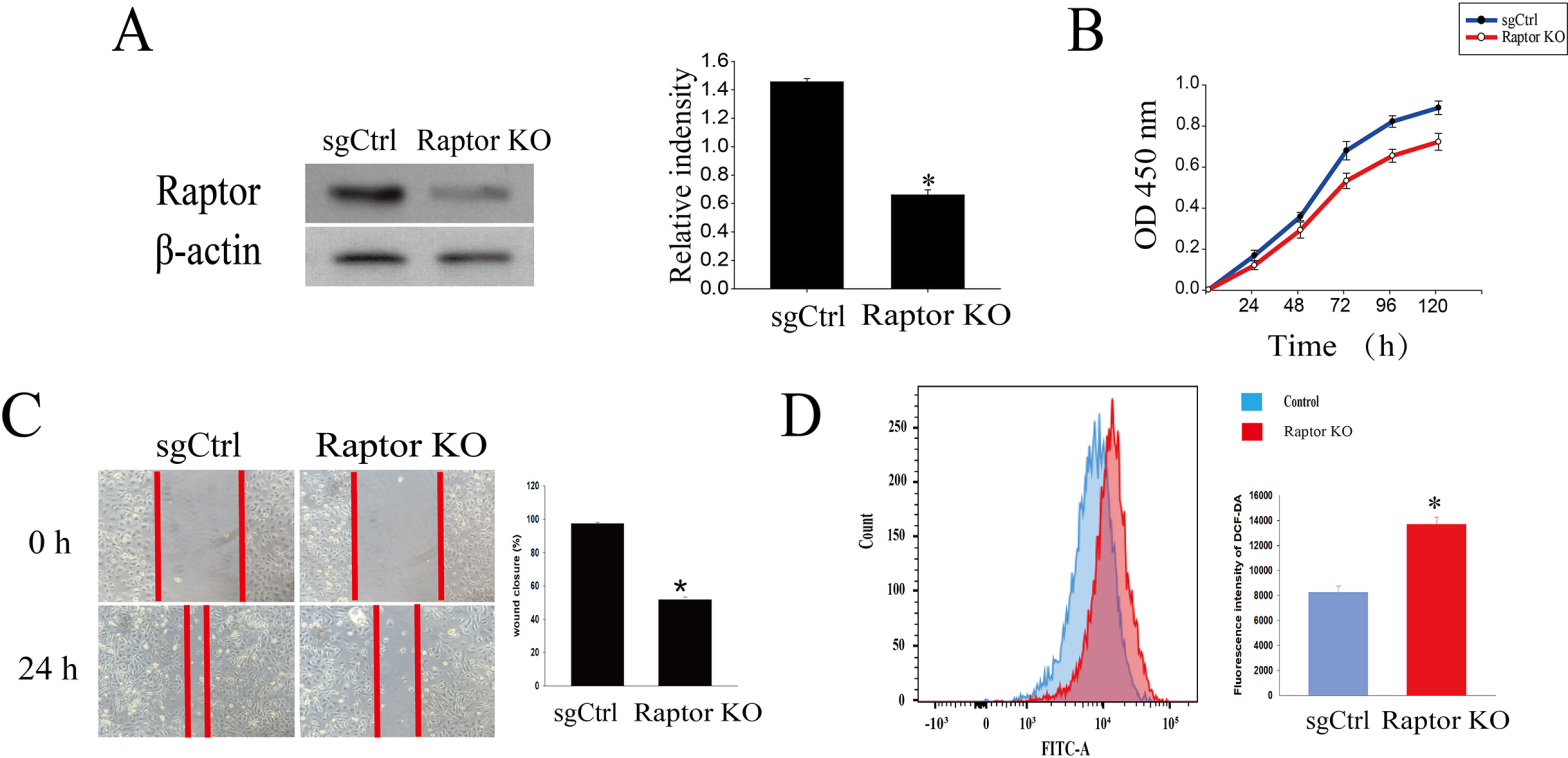
Raptor deletion increased ROS accumulation and inhibited cell viability and migration in SKOV3 cells. (A) The protein bands of Raptor and the relative density ratios of Raptor protein normalized to actin in Raptor KO SKOV3 cells. (B) The cell viability of SKOV3 and Raptor KO SKOV3 cells. (C) The cell migration of SKOV3 and Raptor KO SKOV3 cells. (D) The ROS level in SKOV3 and Raptor KO SKOV3 cells. Mean ± SD, *n* = 3, *P < 0.05 *vs* control group.

It was reported that cardamonin markedly decreased the expression of Raptor, revealing an indispensable role of Raptor in regulation of mTORC1 activity by cardamonin (Shi et al., 2018b). Here, we tested whether Raptor also participated in cardamonin-induced oxidative stress through ERK1/2 and (or) mTORC1 pathways. Raptor was knocked out (KO) by CRISPR-Cas9 in SKOV3 cells (Fig. 6A). Cell viability and migration were significantly inhibited in Raptor deletion cells (Figs. 6B and 6C). However, the production of intracellular ROS was increased in Raptor KO SKOV3 cells (Fig. 6D).

**Figure 7:**
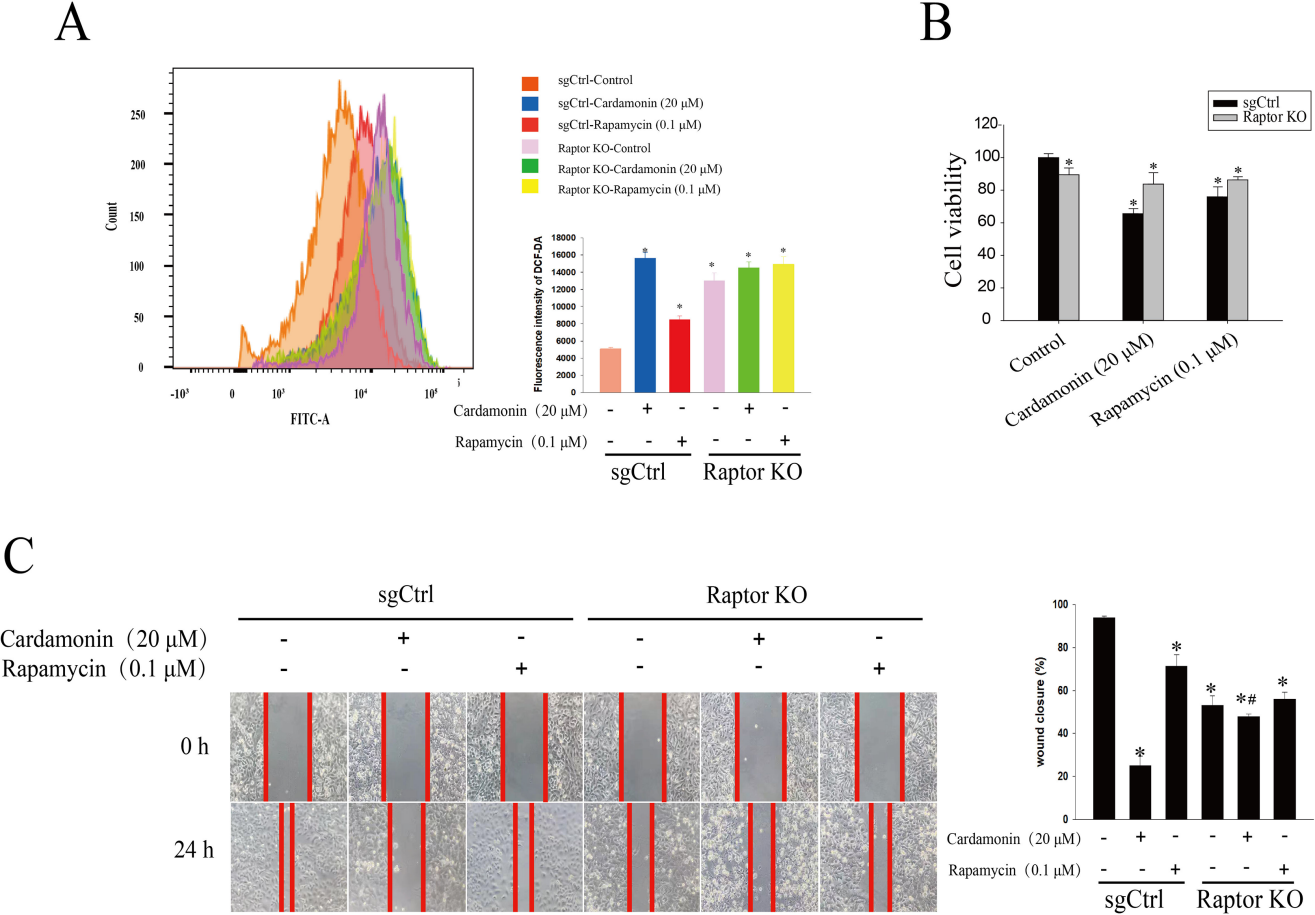
Raptor mediated the anti-proliferation of cardamonin through ROS induction. SKOV3 and Raptor KO SKOV3 cells were treated with cardamonin (20 μM) or rapamycin (0.1 μM) for 24 h, respectively. (A) The ROS level in SKOV3 and Raptor KO SKOV3 cells. (B) The cell viability of SKOV3 and Raptor KO SKOV3 cells. (C) The cell migration of SKOV3 and Raptor KO SKOV3 cells. Mean ± SD, *n* = 3, *P < 0.05 *vs* control group, *^#^P* < 0.05 *vs* sgCtrl groups.

Raptor KO and WT SKOV3 cells were treated with cardamonin (20 μM) and rapamycin (0.1 μM), respectively, and the ROS level was drastically increased in Raptor KO groups, suggesting a potential role of Raptor in ROS induction (Fig. 7A). Consistently, cell viability was significantly inhibited in Raptor KO SKOV3 cells, and the inhibitory effect of cardamonin on Raptor KO SKOV3 cells was attenuated (Fig. 7B). Similar results were observed in wound healing assay (Fig. 7C).

**Figure 8:**
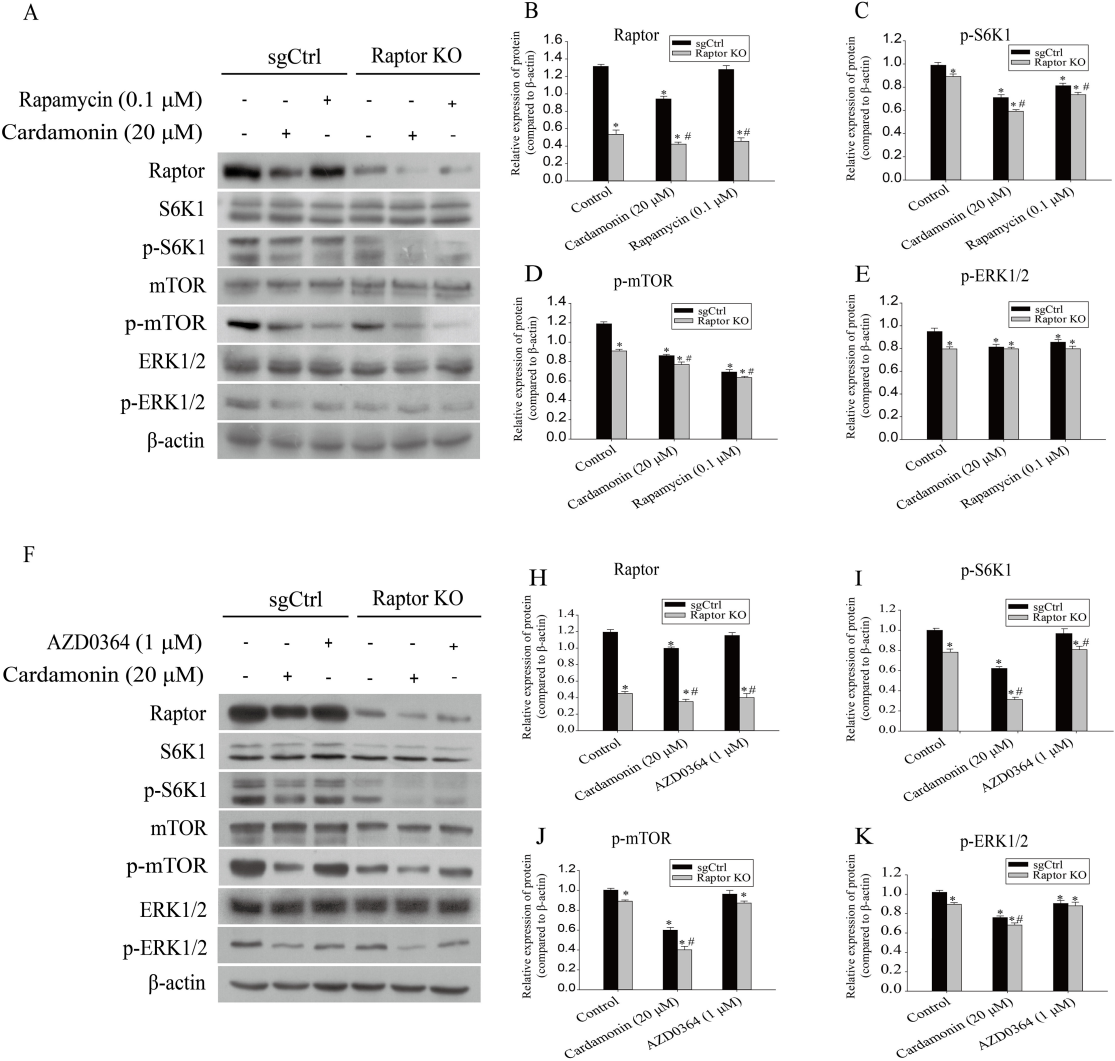
Raptor coupled mTORC1 and ERK1/2 inhibition by cardamonin with oxidative stress induction. SKOV3 and Raptor KO SKOV3 cells were treated with cardamonin (20 μM) or rapamycin (0.1 μM) or AZD0364 (1 μM) for 24 h, respectively. Then, total protein was extracted for Western blotting analysis. (A and F) The protein bands of Raptor, S6K1, p-S6K1, mTOR, p-mTOR, ERK1/2, p-ERK1/2 and actin in two cell lines. (B–E and H–K) The relative density ratios of Raptor, p-S6K1, p-mTOR and p-ERK1/2 protein were normalized to actin in two cell lines. For all panels, error bars are presented as the mean ± SD, *n* = 3, *P < 0.05 *vs* control group, *^#^P* < 0.05 *vs* sgCtrl groups.

Next, the related proteins expression of mTOR and ERK1/2 pathways were examined in Raptor KO SKOV3 cells. Results showed that the expression of p-S6K1 (Thr389) and p-mTOR (Ser2448) was decreased by cardamonin and rapamycin. Similarly, the expression of p-ERK1/2 (Thr202/Tyr204) was also inhibited in cardamonin, rapamycin and ERK1/2 inhibitor AZD0364 groups. Notably, the inhibitory effect of cardamonin on the expression of p-S6K1, p-mTOR and p-ERK1/2 was weakened in Raptor KO cells. Interestingly, the total protein expression of Raptor was only decreased by cardamonin (Fig. 8).

## Discussion

Ovarian cancer is a common gynecologic malignancy with high mortality. Most patients received optimal debulking combined with platinum-based chemotherapy and obtained a good outcome during initial period. Unfortunately, it has been reported that a majority of patients will relapse and eventually develop platinum resistance (Torre et al., 2018; Webber et al., 2019). Therefore, it is of great clinical significance to develop a potential target drug with low toxicity for ovarian cancer therapy.

As a matter of fact, drugs targeting to ROS production is a new strategy for cancer therapy. ROS plays an essential role in cell progression such as cell cycle, cell differentiation and cell death (Kumari et al., 2018). Moderate level of ROS can provide a pro-survival signal to cancer cells. As the present study showed, the viability of ovarian cancer cells was slightly reduced by NAC. However, beyond the steady-state level of intracellular ROS, either shortage or surplus, will damage the growth of tumor cells. So tumor cells will undergo metabolic reprogramming for immortal proliferation. The malfunction of metabolism and mitochondria may elevate oxidative stress and deliciated redox balance, suggesting that targeting redox balance in tumor cells has potential therapeutic benefits. The role of cardamonin in regulation of redox homeostasis has been studied previously. Surprisingly, cardamonin exerted two opposite effects in ROS accumulation. Cardamonin exhibited antioxidant activity by up-regulating of Nrf2 and NF-κB, and then prevented cells from oxidative stress and inflammatory damage in cardiomyocytes (Qi et al., 2020; Tan et al., 2021); on the other hand, cardamonin enhanced mitochondrial oxidative stress through inhibiting HIF-1α or NF-κB, which ultimately inhibits the tumor cell proliferation and migration (Jin et al., 2019; Li et al., 2017). The present study showed that cardamonin exerts anti-cancer effects partly through inducing ROS in agreement with most common chemotherapeutics such as platinum and taxanes. We have previously demonstrated that cardamonin induced apoptosis and autophagy in ovarian cancer cells, and changes in ROS was proved to be a key factor in inducing apoptosis and autophagy (Li et al., 2022; Shi et al., 2018a). Studies also have shown that autophagy stimulated by external factors might related to apoptosis through relieving environmental stress (Adams & Cory, 2007), providing ATP, activating caspase (Bovellan et al., 2010) and degradation of damaged organelles (Kraft, Peter & Hofmann, 2010). Therefore, it is of great significance to further study whether the inhibition of cardamonin on ovarian cancer cell viability is related to ROS mediated apoptosis and cell autophagy.

Consistent with the ROS inducer BAY11-7082, cardamomin significantly induced intracellular ROS levels, which may account for its inhibition of cell viability and migration. The anti-cancer effect of cardamonin through deprivation of the redox balance was further verified by scavenging and inducing intracellular ROS using NAC and Glu, respectively. Notably, cardamonin exhibited strong inhibition on the migration of SKOV3 cells while showed little effect on that of A2780 cells. In addition, neither NAC nor Glu affected the migration of A2780 cells, which may be related to the low metastatic capacity of A2780 cells and the low sensitivity of A2780 cells to changes in ROS levels (Zhao et al., 2016). Besides, different cell lines and incubation time should be taken into account, study has shown that, A2780 cell mobility only showed a subtle difference when incubated for 24 h, but significantly changed when incubated for 48 h. However, there was a noticeable change in migration of SKOV3 cells after incubated for 24 h (Wang et al., 2022). Taken together, it is demonstrated that ROS production or scavenging plays a crucial role in chemotherapy responses in ovarian cancer cells. Furthermore, different signal transduction pathways might be involved in the regulation of ROS by cardamonin under certain circumstance. It is reported that cardamonin could inhibit the expression of HIF-1α and subsequently enhanced mitochondrial oxidative phosphorylation and ROS accumulation, which finally induced apoptosis in breast cancer cells (Jin et al., 2019). Therefore, whether cardamonin increases ROS in ovarian cancer by targeting mitochondria needs further investigation.

Accumulating evidence indicated that hyper-activation of ERK1/2 might regulate cell cycle progression and tumorigenesis (Cheng et al., 2021), and ROS-induced ERK activation is related to autophagy-dependent cell death (Hao et al., 2020). ERK1/2 senses ROS signaling by different mechanisms including Ras activation, MAPK kinases (MAPKK) activation or MAPK phosphatases inactivation. Studies have also shown that inactivation of ERK1/2 might induce the accumulation of ROS by suppression of Nrf2, HO-1 and TrxR, which finally resulted in cell growth inhibition (Jasek-Gajda et al., 2020). Results of proteomic analyses and Western blotting analysis showed that cardamonin had a significant effect on the MAPK signaling pathway. Combined with the results of previous studies, cardamonin-induced oxidative stress and cell death in ovarian cancer cells might be related to the inhibition of ERK1/2 signaling pathway.

Since regulation of ROS level is not sufficient to suppress tumor growth by ERK alone, we further explored whether mTORC1 was related with ROS induction by cardamonin. mTORC1 is a well known positively regulator of malignancy, and it has been demonstrated that ROS-dependent inhibition of mTOR pathway might result in autophagy, apoptosis and cell death in ovarian cancer cells (Shi et al., 2018b; Wang et al., 2021b). Rapamycin, a classic inhibitor of mTORC1, has been reported to increase ROS generation and finally induce tumor cell apoptosis and death (Zimmerman et al., 2020). Conversely, activation of the PI3K/Akt/mTOR pathway was found to protect A549 cells from H_2_O_2_-induced oxidative injury and apoptosis (Ma et al., 2021). In consistent with our previous studies, cardamonin and rapamycin inhibited the activity of mTORC1, which could be rescued by mTOR agonist MHY1485. These findings indicated that oxidative stress induced by cardamonin partly through inhibiting of mTORC1 pathway in ovarian cancer cells.

Furthermore, we investigated the role of ERK1/2 and mTORC1 in cardamonin-induced oxidative stress, based on the potentially link between these two pathways (Li et al., 2011). Previously, we confirmed that cardamonin suppressed the proliferation by mTORC1 inhibition through down-regulation the expression of Raptor in ovarian cancer cells (Shi et al., 2018b). It is interestingly to clarify whether Raptor is a critical protein in mediating the ROS production by cardamonin and whether Raptor is a linker between ERK1/2 and mTOR signal pathways. Phosphorylation of Raptor on multiple sites is essential for its fine-tuning effect on mTORC1 signaling (Antonia et al., 2019; Foster et al., 2010) and the translocation of mTOR to lysosomes (Sancak et al., 2008). Over-expression of Raptor was related to drug resistance (Shchegolev et al., 2020). As expected, deletion of Raptor remarkably inhibited cell viability and migration, accompanied by an increased intracellular ROS level. In addition, the induction of ROS and subsequently inhibition on cell growth were weakened by cardamonin in Raptor KO SKOV3 cells, indicating an indispensable role of Raptor in anti-cancer effect of cardamonin on ovarian cancer.

It was reported that Raptor bound to SOHC2, an agonist of RAS upstream of ERK1/2, thereby blocking the ERK1/2 pathway and cell proliferation. SHOC2-Raptor interaction also triggered the negative cross-talk between RAS-ERK and mTORC1 pathways (Xie et al., 2019). We tested the related proteins expression of mTOR and ERK1/2 pathway upon Raptor depletion, and the results showed that a significantly decrease of p-mTOR, p-S6K1 and p-ERK1/2 was observed in Raptor KO SKOV3 cells. As a control, AZD0364 decreased expression of p-ERK1/2 and its inhibitory effect was unaffected by Raptor depletion. The findings supported the speculation that Raptor is the particular protein regulated by cardamonin, and partially mediated the induction of oxidative stress through mTOR and ERK1/2 pathways. In addition, regarding Raptor as a scaffold for mTOR activity is an over-simplification, it was reported that mTORC1-independent Raptor also makes sense in cell metabolism and cancer progression (Van Nostrand et al., 2020). Generally, whether Raptor regulates ERK1/2 in an mTORC1-dependent or mTORC1-independent manner and by which mechanism Raptor senses ROS signaling requires further investigation.

## Conclusion

In conclusion, the present study elucidates that cardamonin controls cell proliferation through inducing oxidative stress and reveals an underlying cross talk between mTORC1 and ERK1/2 pathways *via* Raptor, providing a promising target for novel drug development and ovarian cancer therapy.

## Significance

The present research has demonstrated that the inhibition of cardamonin on ovarian cancer *via* inducing oxidative stress. The underlying mechanism was associated with down-regulating Raptor expression and regulation of the mTORC1-ERK1/2 pathway. This study provides a promising target for ovarian cancer therapy.

## Supplemental Information

10.7717/peerj.15498/supp-1 Supplemental Information 1 cardamonin inhibited the proliferation of ovarian cancer cells.

10.7717/peerj.15498/supp-2 Supplemental Information 2 Cardamonin induced the ROS in ovarian cancer cells.

10.7717/peerj.15498/supp-3 Supplemental Information 3 Cardamonin induced the ROS in ovarian cancer cells.

10.7717/peerj.15498/supp-4 Supplemental Information 4 Cardamonin inhibited the migration of ovarian cancer cells.

10.7717/peerj.15498/supp-5 Supplemental Information 5 Cardamonin inhibited the migration of ovarian cancer cells.

10.7717/peerj.15498/supp-6 Supplemental Information 6 Cardamonin inhibited the migration of ovarian cancer cells.

10.7717/peerj.15498/supp-7 Supplemental Information 7 Cardamonin regulated the expression of relative proteins in ovarian cancer cells.

10.7717/peerj.15498/supp-8 Supplemental Information 8 Wound healing assay quantifying.

## Additional Information and Declarations

### Data Availability

The following information was supplied regarding data availability: The raw measurements are available in the Supplemental Files. The raw data shows cardamonin inhibited the proliferation of ovarian cancer SKOV3 and A2780 cells in dose-dependent way and accompanied by ROS induction. Two cell lines were then used to investigate the mechanism that cardamonin inhibition of ovarian cancer cell growth *via* induction of oxidative stress.
