# Supplementary material for: Raptor couples mTORC1 and ERK1/2 inhibition by cardamonin with oxidative stress induction in ovarian cancer cells
Source: PeerJ. 2023 Jun 7;11:e15498. doi: 10.7717/peerj.15498 (PMC10257395; doi:10.7717/peerj.15498)
Supplement: Supplemental Information 2 [file peerj-11-15498-s002.pdf]

BD FACSDiva 8.0.3

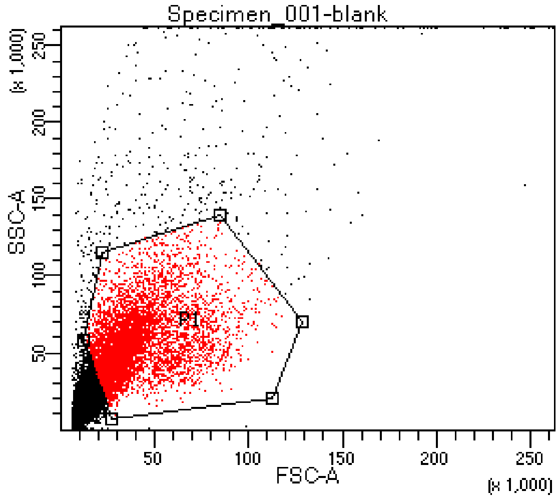

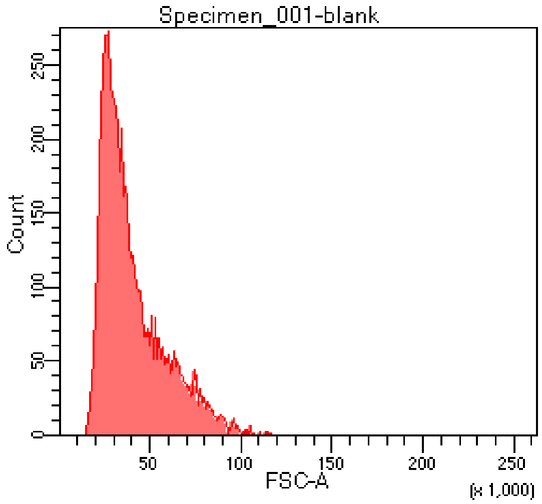

Experiment Name:	20200203 ROS A2780
Specimen Name:	Specimen_001
Tube Name:	blank
Record Date:	Feb 3, 2021 2:17:04 PM
$OP:	Administrator
GUID:	8768084a-1a61-4e06-90ea-39...

Population	#Events	%Parent	FSC-A Mean
P1	6,598	29.8	38,735

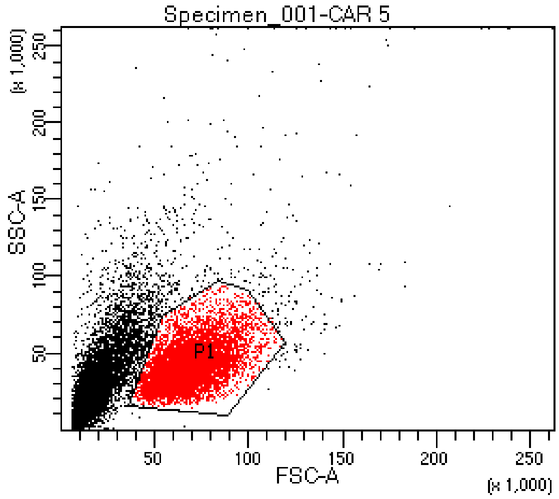

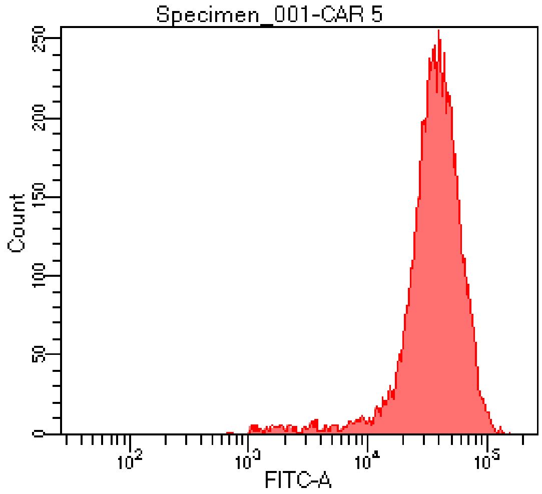

Experiment Name:	20200203 ROS A2780
Specimen Name:	Specimen_001
Tube Name:	CAR 5
Record Date:	Feb 3, 2021 2:25:05 PM
$OP:	Administrator
GUID:	7ddc9ed8-7670-449e-8bb2-76b...

Population	#Events	%Parent	FITC-A Mean
P1	7,350	40.1	38,016

BD FACSDiva 8.0.3

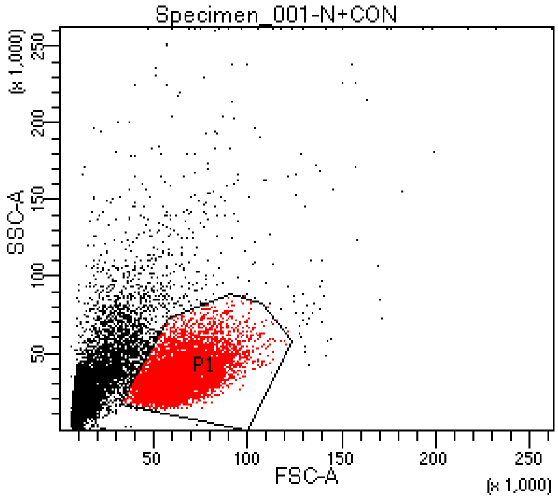

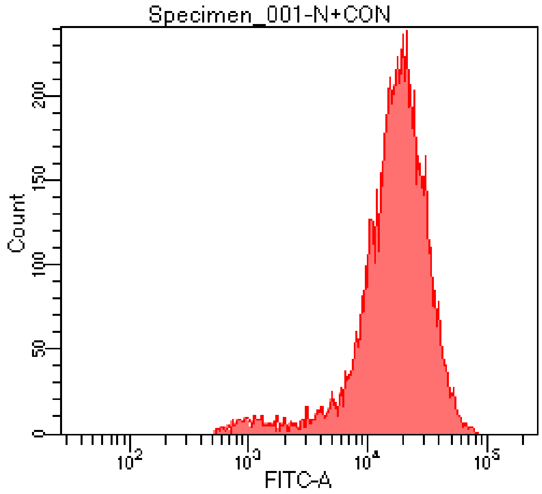

Experiment Name:	20200203 ROS A2780
Specimen Name:	Specimen_001
Tube Name:	N+CON
Record Date:	Feb 3, 2021 2:30:29 PM
$OP:	Administrator
GUID:	6e86711b-176f-4285-aef2-f9bfa...

Population	#Events	%Parent	FITC-A Mean
P1	8,012	47.2	18,752

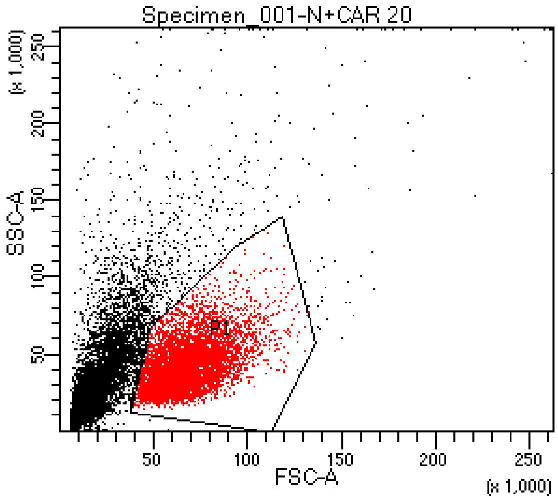

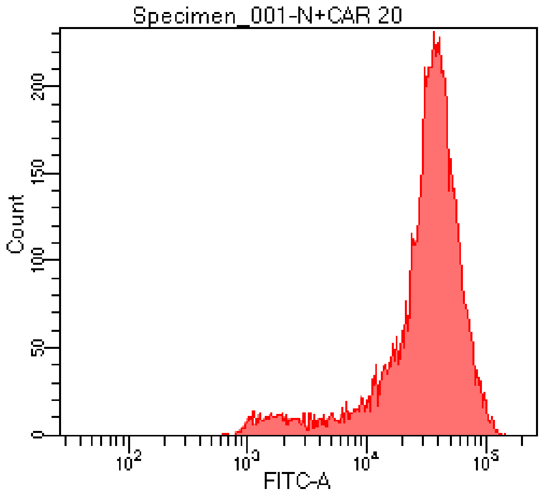

Experiment Name:	20200203 ROS A2780
Specimen Name:	Specimen_001
Tube Name:	N+CAR 20
Record Date:	Feb 3, 2021 2:32:36 PM
$OP:	Administrator
GUID:	bed97aff-199a-4def-a685-6556...

Population	#Events	%Parent	FITC-A Mean
P1	7,139	37.9	34,804

BD FACSDiva 8.0.3

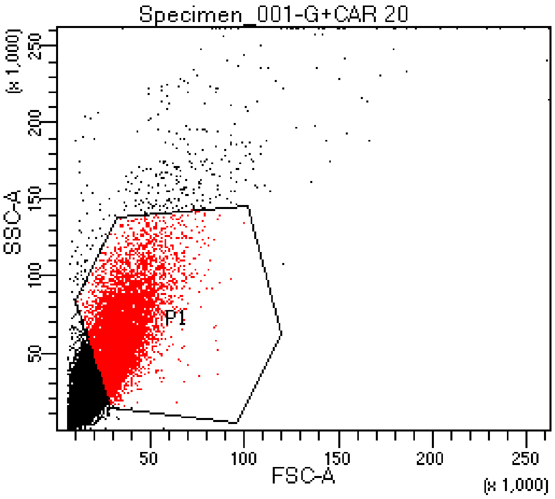

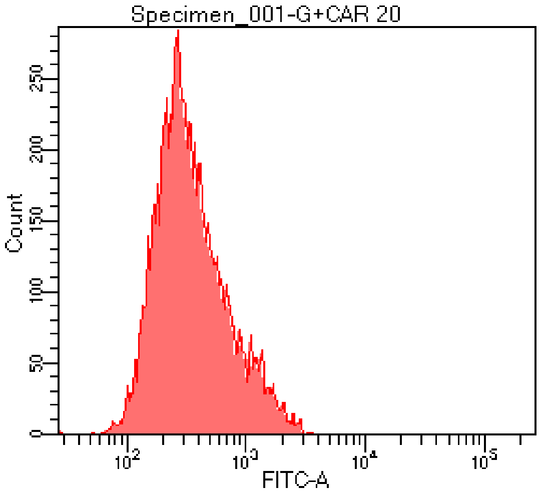

Experiment Name:	20200203 ROS A2780
Specimen Name:	Specimen_001
Tube Name:	G+CAR 20
Record Date:	Feb 3, 2021 2:37:22 PM
$OP:	Administrator
GUID:	4fe44348-7489-46fb-8c73-8e06...

Population	#Events	%Parent	FITC-A Mean
P1	10,114	22.3	453

BD FACSDiva 8.0.3

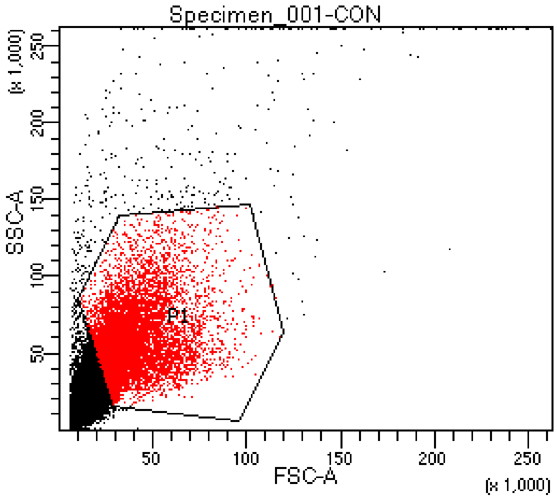

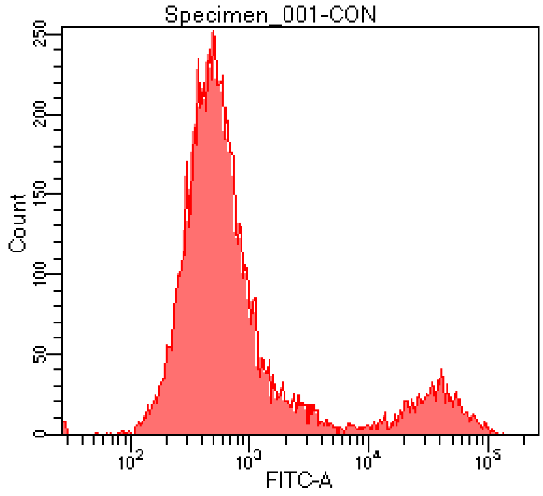

Experiment Name:	20200203 ROS A2780
Specimen Name:	Specimen_001
Tube Name:	CON
Record Date:	Feb 3, 2021 2:23:39 PM
$OP:	Administrator
GUID:	48c431cc-54c8-4a79-8bfa-48d4...

Population	#Events	%Parent	FITC-A Mean
P1	10,000	23.1	5,010

BD FACSDiva 8.0.3

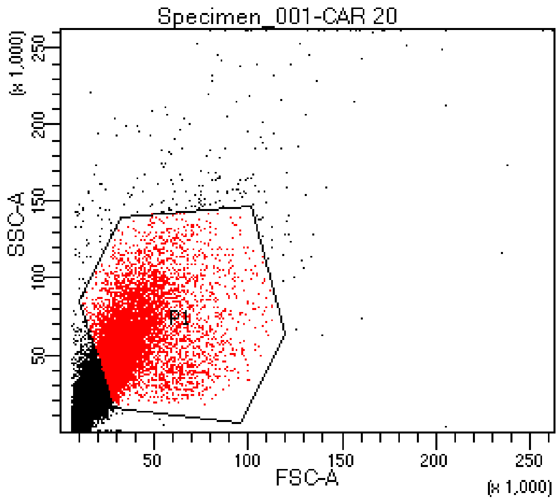

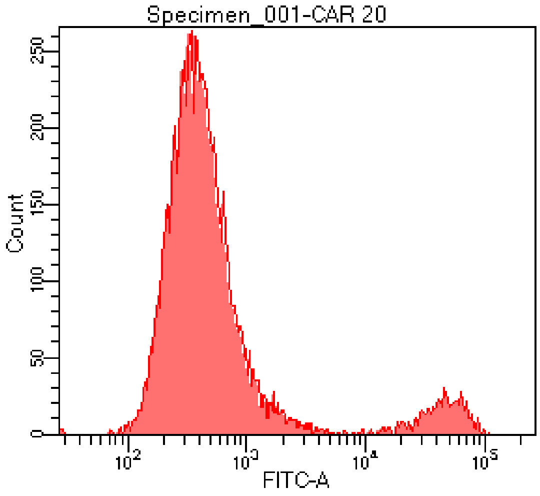

Experiment Name:	20200203 ROS A2780
Specimen Name:	Specimen_001
Tube Name:	CAR 20
Record Date:	Feb 3, 2021 2:28:33 PM
$OP:	Administrator
GUID:	5a426e17-4af8-4aa2-8974-e80...

Population	#Events	%Parent	FITC-A Mean
P1	10,000	20.9	3,941

BD FACSDiva 8.0.3

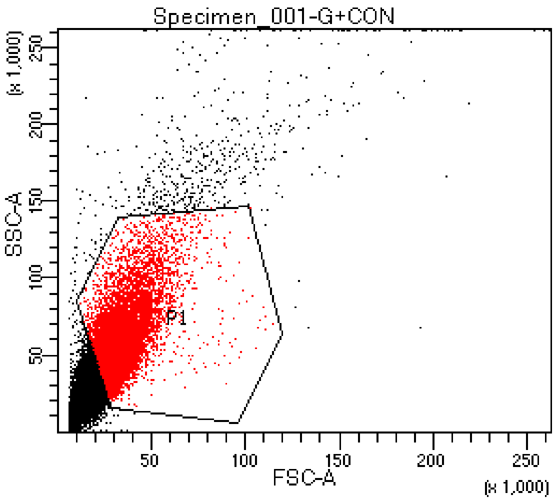

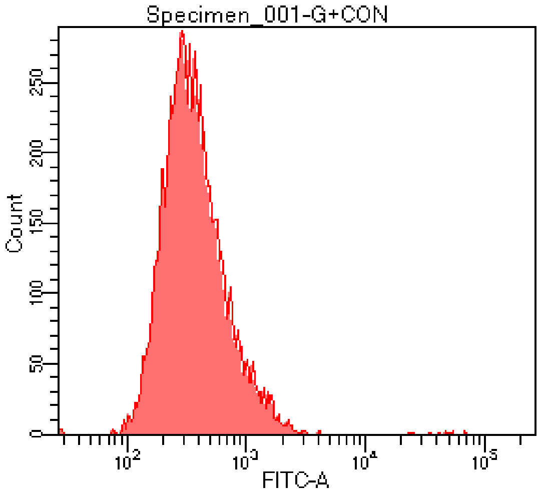

Experiment Name:	20200203 ROS A2780
Specimen Name:	Specimen_001
Tube Name:	G+CON
Record Date:	Feb 3, 2021 2:35:25 PM
$OP:	Administrator
GUID:	7a2358dc-845b-41e3-8535-6fe...

Population	#Events	%Parent	FITC-A Mean
P1	10,000	25.2	656
