# Supplementary material for: Raptor couples mTORC1 and ERK1/2 inhibition by cardamonin with oxidative stress induction in ovarian cancer cells
Source: PeerJ. 2023 Jun 7;11:e15498. doi: 10.7717/peerj.15498 (PMC10257395; doi:10.7717/peerj.15498)
Supplement: Supplemental Information 3 [file peerj-11-15498-s003.pdf]

BD FACSDiva 8.0.3

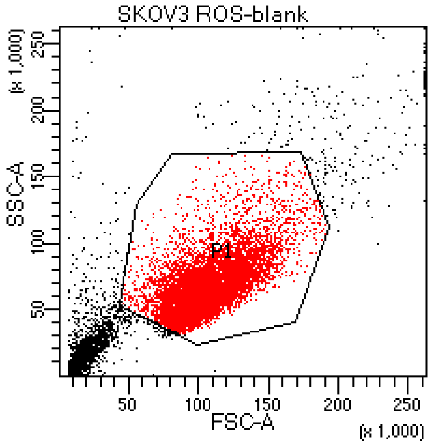

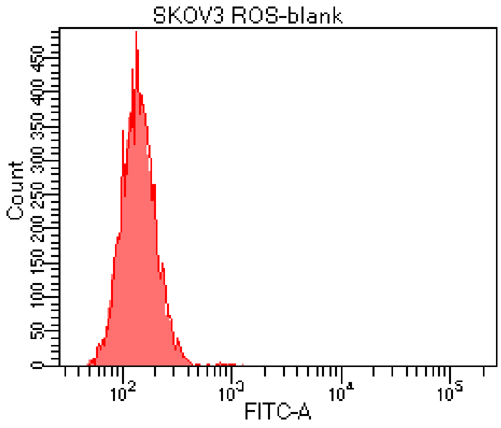

Experiment Name:	20210203 ROS
Specimen Name:	SKOV3 ROS
Tube Name:	blank
Record Date:	Feb 3, 2021 1:52:53 PM
$OP:	Administrator
GUID:	a4fe4dc0-c53e-4f8d-8cdb-cefa0...

Population	#Events	%Parent	FITC-A Mean
P1	10,000	61.6	170

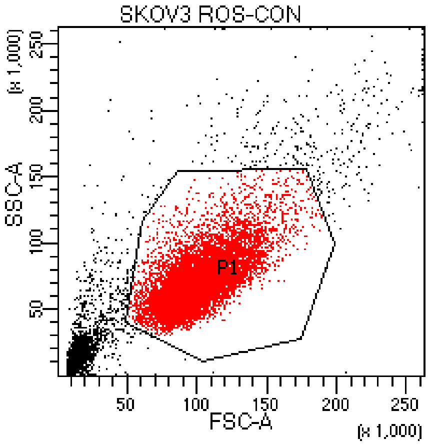

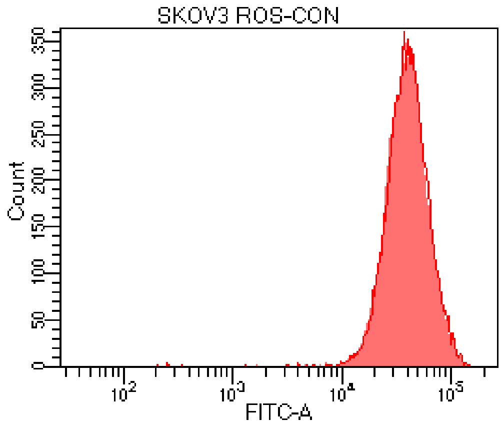

Experiment Name:	20210203 ROS
Specimen Name:	SKOV3 ROS
Tube Name:	CON
Record Date:	Feb 3, 2021 1:54:26 PM
$OP:	Administrator
GUID:	b1673093-4725-4e14-9de6-e9...

Population	#Events	%Parent	FITC-A Mean
P1	10,000	67.8	41,085

BD FACSDiva 8.0.3

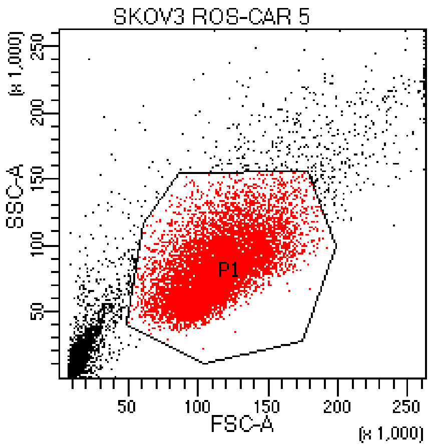

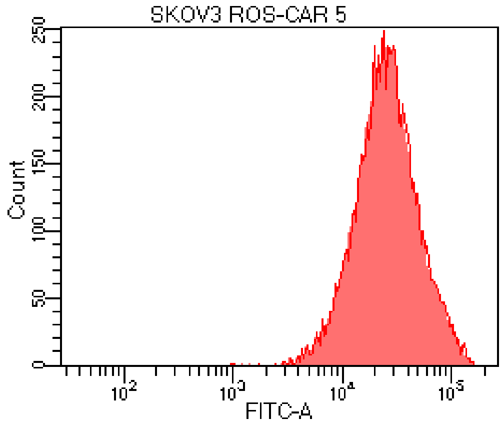

Experiment Name: 20210203 ROS
Specimen Name: SKOV3 ROS
Tube Name: CAR 5
Record Date: Feb 3, 2021 1:57:48 PM
$OP: Administrator
GUID: 03a7c985-8096-4f4c-8cca-1d47...

Population	#Events	%Parent	FITC-A Mean
P1	10,000	64.8	29,986

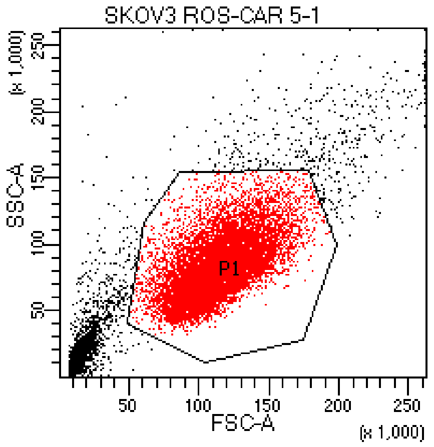

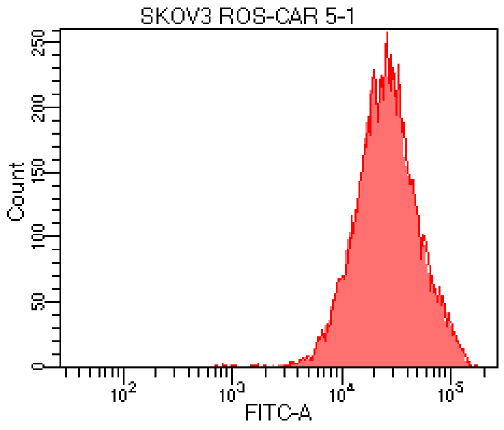

Experiment Name: 20210203 ROS
Specimen Name: SKOV3 ROS
Tube Name: CAR 5-1
Record Date: Feb 3, 2021 1:58:32 PM
$OP: Administrator
GUID: ab1ca5e6-b4f6-4e4b-9fb4-caaa...

Population	#Events	%Parent	FITC-A Mean
P1	10,000	64.7	30,340

BD FACSDiva 8.0.3

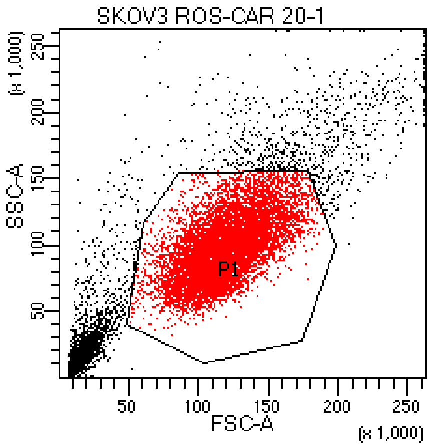

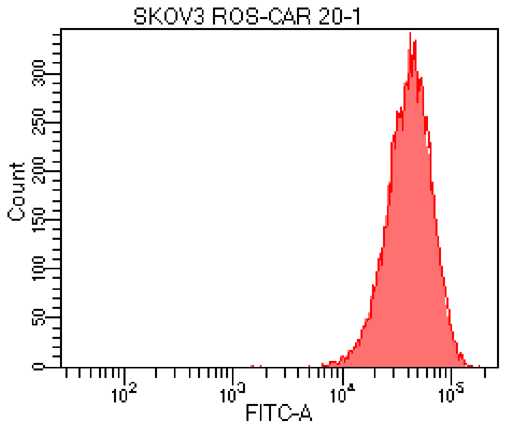

Experiment Name:	20210203 ROS
Specimen Name:	SKOV3 ROS
Tube Name:	CAR 20-1
Record Date:	Feb 3, 2021 2:01:02 PM
$OP:	Administrator
GUID:	da6bbd61-d62a-49df-b6ea-dc6...

Population	#Events	%Parent	FITC-A Mean
P1	10,000	58.3	42,968

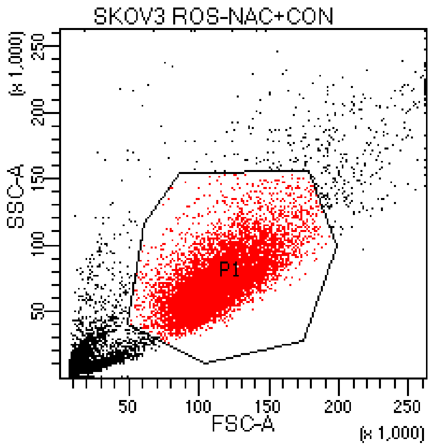

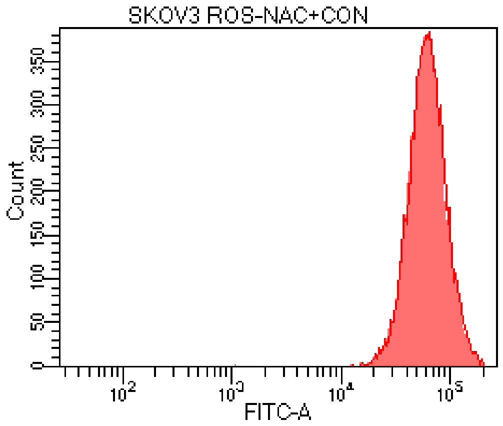

Experiment Name:	20210203 ROS
Specimen Name:	SKOV3 ROS
Tube Name:	NAC+CON
Record Date:	Feb 3, 2021 2:03:05 PM
$OP:	Administrator
GUID:	246fdb10-5132-4d6b-a27b-7fd4...

Population	#Events	%Parent	FITC-A Mean
P1	10,000	63.5	63,344

BD FACSDiva 8.0.3

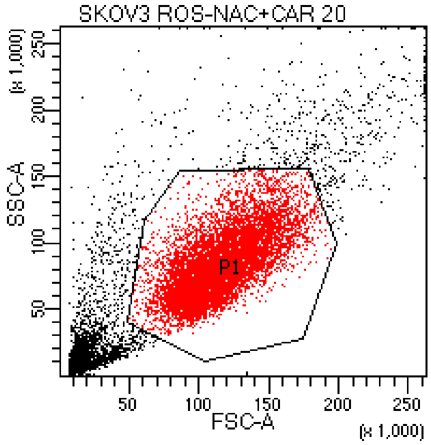

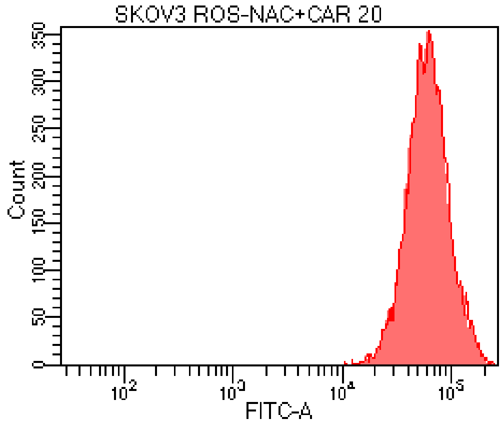

Experiment Name:	20210203 ROS
Specimen Name:	SKOV3 ROS
Tube Name:	NAC+CAR 20
Record Date:	Feb 3, 2021 2:05:37 PM
$OP:	Administrator
GUID:	eb5047fb-cdc2-4166-9bf5-50f7...

Population	#Events	%Parent	FITC-A Mean
P1	10,000	68.4	63,778

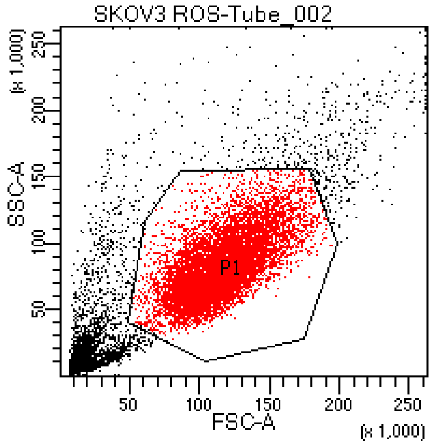

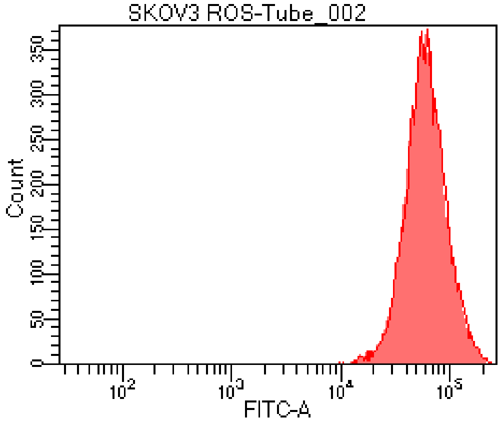

Experiment Name:	20210203 ROS
Specimen Name:	SKOV3 ROS
Tube Name:	Tube_002
Record Date:	Feb 3, 2021 2:07:21 PM
$OP:	Administrator
GUID:	2a43f83c-f5ef-44f6-9018-ace43...

Population	#Events	%Parent	FITC-A Mean
P1	10,000	67.5	62,944

BD FACSDiva 8.0.3

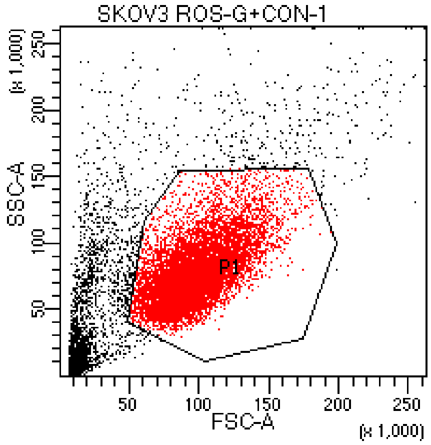

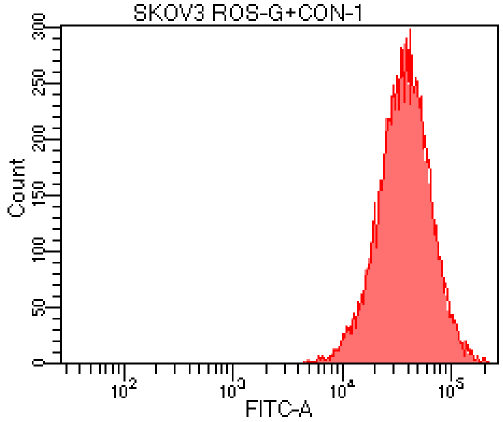

Experiment Name:	20210203 ROS
Specimen Name:	SKOV3 ROS
Tube Name:	G+CON-1
Record Date:	Feb 3, 2021 2:09:34 PM
$OP:	Administrator
GUID:	ae244ba6-b484-4c6d-909b-17e...

Population	#Events	%Parent	FITC-A Mean
P1	10,000	71.2	41,122

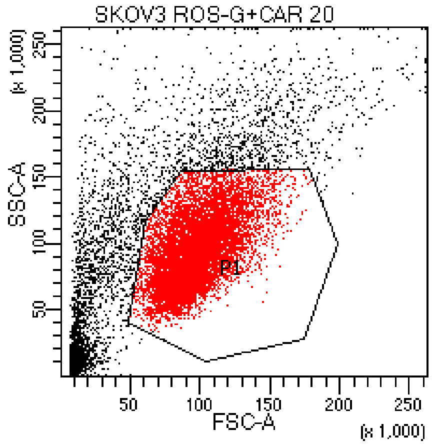

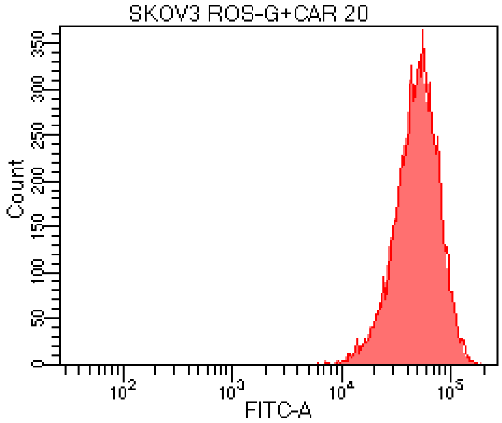

Experiment Name:	20210203 ROS
Specimen Name:	SKOV3 ROS
Tube Name:	G+CAR 20
Record Date:	Feb 3, 2021 2:10:36 PM
$OP:	Administrator
GUID:	aeeb7624-105f-442a-a6bf-7cdf...

Population	#Events	%Parent	FITC-A Mean
P1	10,000	66.4	51,355

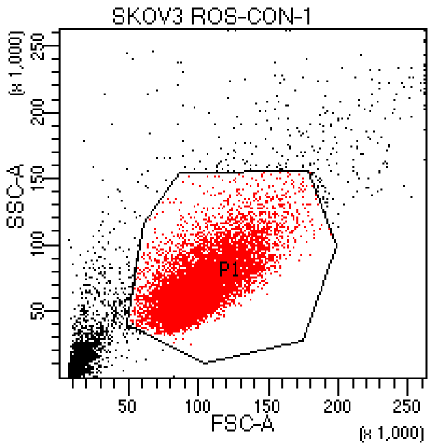

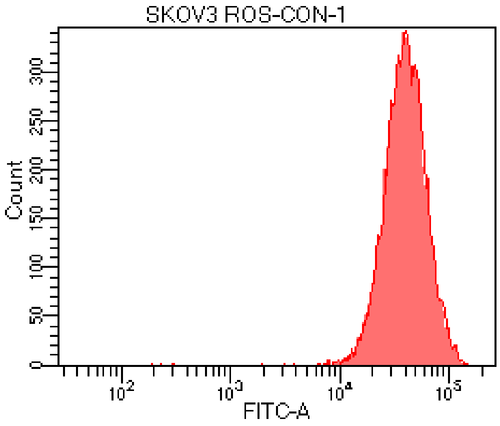

Experiment Name:	20210203 ROS
Specimen Name:	SKOV3 ROS
Tube Name:	CON-1
Record Date:	Feb 3, 2021 1:55:07 PM
$OP:	Administrator
GUID:	1f59dce8-5e90-4e18-a2e4-54c...

Population	#Events	%Parent	FITC-A Mean
P1	10,000	66.5	41,242

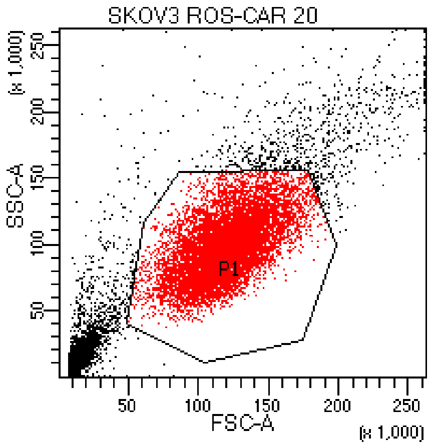

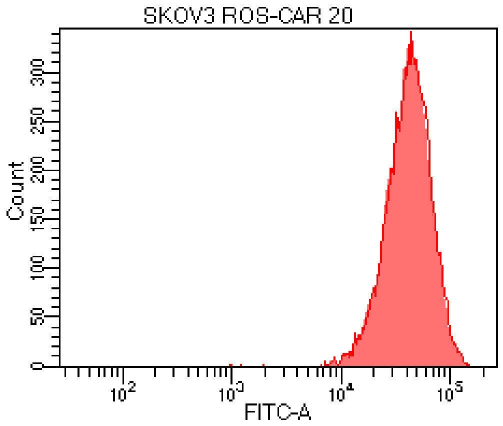

Experiment Name:	20210203 ROS
Specimen Name:	SKOV3 ROS
Tube Name:	CAR 20
Record Date:	Feb 3, 2021 2:00:16 PM
$OP:	Administrator
GUID:	623f43d7-223f-4e39-b563-65e5...

Population	#Events	%Parent	FITC-A Mean
P1	10,000	59.2	43,349

BD FACSDiva 8.0.3

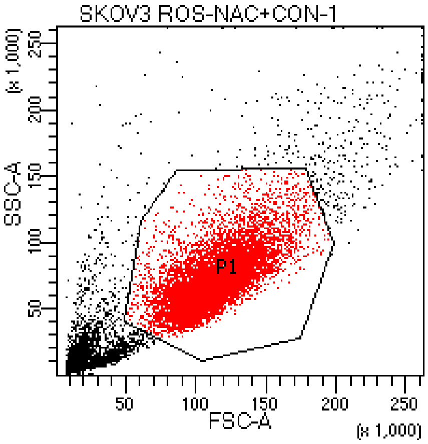

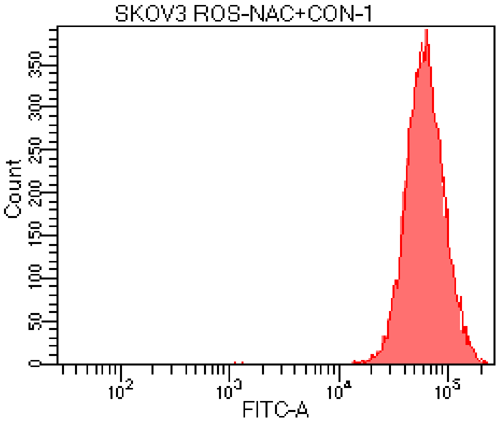

Experiment Name:	20210203 ROS
Specimen Name:	SKOV3 ROS
Tube Name:	NAC+CON-1
Record Date:	Feb 3, 2021 2:03:51 PM
$OP:	Administrator
GUID:	786f1c78-81cc-47c3-b2d2-ec18...

Population	#Events	%Parent	FITC-A Mean
P1	10,000	63.6	63,803

BD FACSDiva 8.0.3

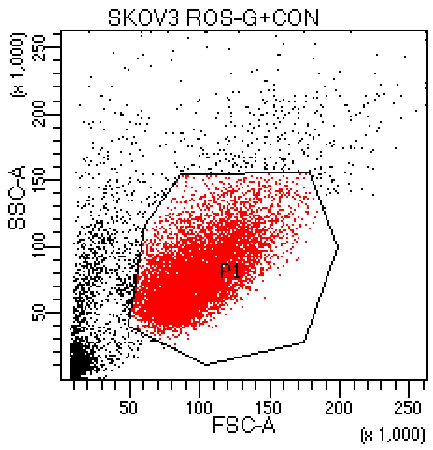

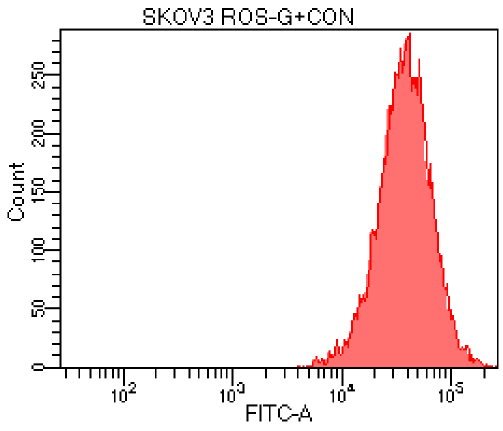

Experiment Name:	20210203 ROS
Specimen Name:	SKOV3 ROS
Tube Name:	G+CON
Record Date:	Feb 3, 2021 2:08:51 PM
$OP:	Administrator
GUID:	43776e47-de10-41dd-8eb4-c01...

Population	#Events	%Parent	FITC-A Mean
P1	10,000	73.4	41,770

BD FACSDiva 8.0.3

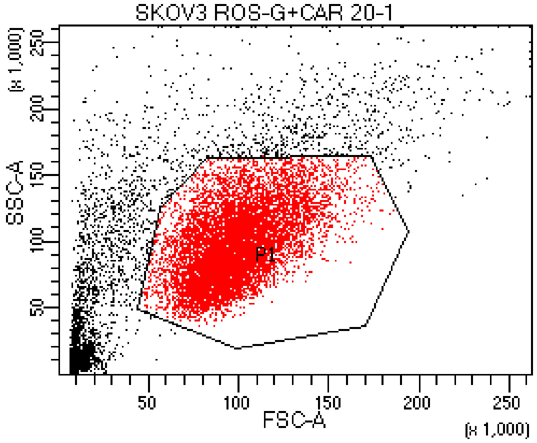

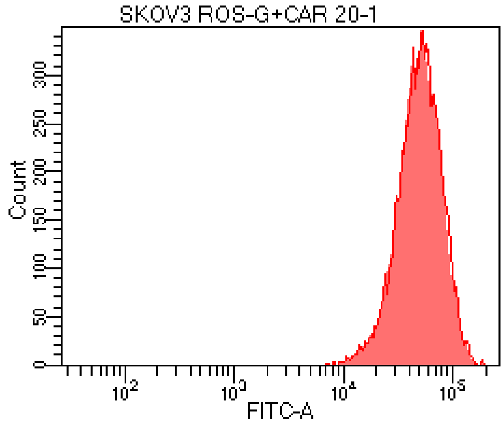

Experiment Name:	20210203 ROS
Specimen Name:	SKOV3 ROS
Tube Name:	G+CAR 20-1
Record Date:	Feb 3, 2021 2:11:25 PM
$OP:	Administrator
GUID:	6e9e97d5-b082-45fe-809d-ff21...

Population	#Events	%Parent	FITC-A Mean
P1	10,324	68.6	52,071
